# Combined Application of Hyaluronidase and Collagenase for Late-Onset Edema in Periocular Area After Hyaluronic Acid Volume Repositioning: A Six-Case Retrospective Review

**DOI:** 10.7759/cureus.74297

**Published:** 2024-11-23

**Authors:** Desiree Giselle Castelanich, Luis A Parra Hernandéz, Andreina Martinez Amado, Diana A Acevedo, Lina Velásquez, Valentina Dicker, Andrea M Parra Hernandez

**Affiliations:** 1 Dermatology, Argentine Society of Dermatology, Buenos Aires, ARG; 2 Aesthetic Medicine, Sociedad Internacional de Rejuvenecimiento Facial No Quirúrgico (SIRF), Barranquilla, COL; 3 Aesthetic Medicine and Clinical Research, Universidad Militar Nueva Granada, Bogota, COL; 4 Aesthetic Medicine, Universidad del Rosario, Cali, COL; 5 Dermatology, Colombian Society of Dermatology, Cali, COL; 6 Aesthetic Medicine, Universidad del Rosario, Bogota, COL; 7 Oculoplastic Surgery, Sociedad Internacional de Rejuvenecimiento Facial No Quirúrgico (SIRF), Barranquilla, COL

**Keywords:** case report, collagenase, hyaluronic acid, hyaluronidase, late onset edema

## Abstract

Background and objective: Although generally low-risk, hyaluronic acid (HA) dermal fillers can lead to late-onset edema, particularly in the periocular region. This condition typically manifests three to four months post-injection and requires specialized management, usually with hyaluronidase. However, increased use of hyaluronidase has resulted in instances of post-hyaluronidase syndrome, leading to unaesthetic outcomes. This study presents a retrospective case series that utilizes a novel technique combining two enzymes to improve late-onset edema and prevent post-hyaluronidase syndrome development.

Methods: From 2019 to 2024, six patients in our aesthetic clinic received a novel therapeutic approach involving co-administration of 1,500 IU of hyaluronidase and collagenase with a cannula to address late-onset edema in the periocular area.

Results: The combination of high-dose hyaluronidase and low-dose collagenase improved late-onset edema in all patients after a single treatment. Statistical analysis showed a significant improvement in aesthetic scores (P < 0.05), with effect sizes of 0.89 for Hirmand, 1.3 for the Teoxane Infraorbital Hollows Scale (TIOHS), and 1.2 for O'Mahoney’s photo-numeric scale. No post-hyaluronidase syndrome or complications were observed.

Conclusions: This combined technique utilizing 1,500 IU of hyaluronidase and collagenase GH PB220 from Pbserum (Madrid, Spain) effectively achieves significant aesthetic improvements with a high safety profile, offering a promising alternative for managing late-onset edema after HA dermal filler treatments.

## Introduction

The aging process around the eyes involves sagging upper eyelids, noticeable under-eye depressions, and changes in facial bone structure [1]. Cosmetic procedures to refresh the eyelids are well-known for achieving a youthful appearance. However, the periorbital area has a complex anatomy and high vascular risk [2,3]. Hyaluronic acid (HA) fillers are a minimally invasive cosmetic injectable procedure used globally, with a favorable safety profile in most cases. Nevertheless, complications can occur, classified as early, late, or delayed reactions. Based on symptom onset, management options include oral antibiotics (mainly macrolides), incision/drainage, and hyaluronidase injection [2]. Hyaluronidase injection is the gold standard for treating HA complications [4,5]. Periorbital complications can be severe, including blindness, embolism, ptosis, visual alterations, pain, diplopia, ophthalmoplegia, dryness, overfilled syndrome, and the Tyndall effect [2,4]. Less severe complications include bruising (10-75%), color changes (4-7%), contour irregularities (11%), and swelling, fluid, or edema due to mediated or delayed hypersensitivity or hyaluronic acid degradation (15-26%) [6-8].

Hyaluronidase has become a crucial medication in every physician's emergency kit for filler treatments, as it is a soluble enzyme that degrades hyaluronic acid (HA) by hydrolyzing β 1,4-N-acetylglucosaminidic bonds [5,9]. In aesthetics, physicians can use commercial formulations of hyaluronidase, including bovine (bovine testicular hyaluronidase), ovine (ovine testicular hyaluronidase), and human recombinant or bacterial recombinant enzymes, such as Pbserum (Pbserum, Madrid, Spain) from *Streptococcus pyogenes* [5]. Based on their action targets, hyaluronidases can be categorized into three groups: (i) those that manipulate structural tissue characteristics through targeted degradation of hyaluronan [10], (ii) those that modify tissue diffusion properties through targeted hyaluronan degradation [10], and (iii) those that utilize metabolic effects through targeted reduction of hyaluronan chain lengths [10].

The established guidelines recommend a 1:1 ratio of hyaluronidase to the volume of edema in the periocular area. Fifteen hundred units of hyaluronidase are typically diluted in 5 ml of sodium chloride (NaCl) and administered at 500 units per affected area [11]. However, research has demonstrated that the sensitivity of an HA to degradation varies according to the type of reticular product utilized or its molecular weight. Additionally, the brand of hyaluronidase and its origin can cause the volume required to facilitate the complication to vary up to three-fold from one to another. Consequently, it has been challenging to establish a precise quantity or volume of hyaluronidase to employ in all cases necessitating it [12].

While hyaluronidase has a high effectiveness profile in resolving HA-related complications, concerns have recently arisen regarding undesirable outcomes after its application, including pain, irreversible volume loss, and adverse changes in skin appearance. This condition is known as "post-hyaluronidase syndrome" [13]. This condition is reported in approximately 18% of hyaluronidase applications [13]. However, this syndrome's exact mechanism of action is not yet fully understood, and some authors suggest it may be related to the volume and duration of the previously injected filler [13,14]. Enzymatic saturation may occur, replacing HA in the extracellular matrix. Repeated hyaluronidase applications can cause enzymatic saturation, negatively impacting skin quality in the affected area [10]. This risk is particularly significant in the periocular region, where the skin is thinner and more sensitive to volume loss and changes in the extracellular matrix. External factors such as pH, ambient temperature, and tissue type can also influence this risk [15].

This article presents a retrospective case series of six patients who experienced late-onset edema in the periocular area between 2019 and 2024. They were treated with a new technique: applying a higher dose of hyaluronidase in a single session and combining it with collagenase to minimize post-hyaluronidase risk and improve edema.

## Materials and methods

**Figure 1:**
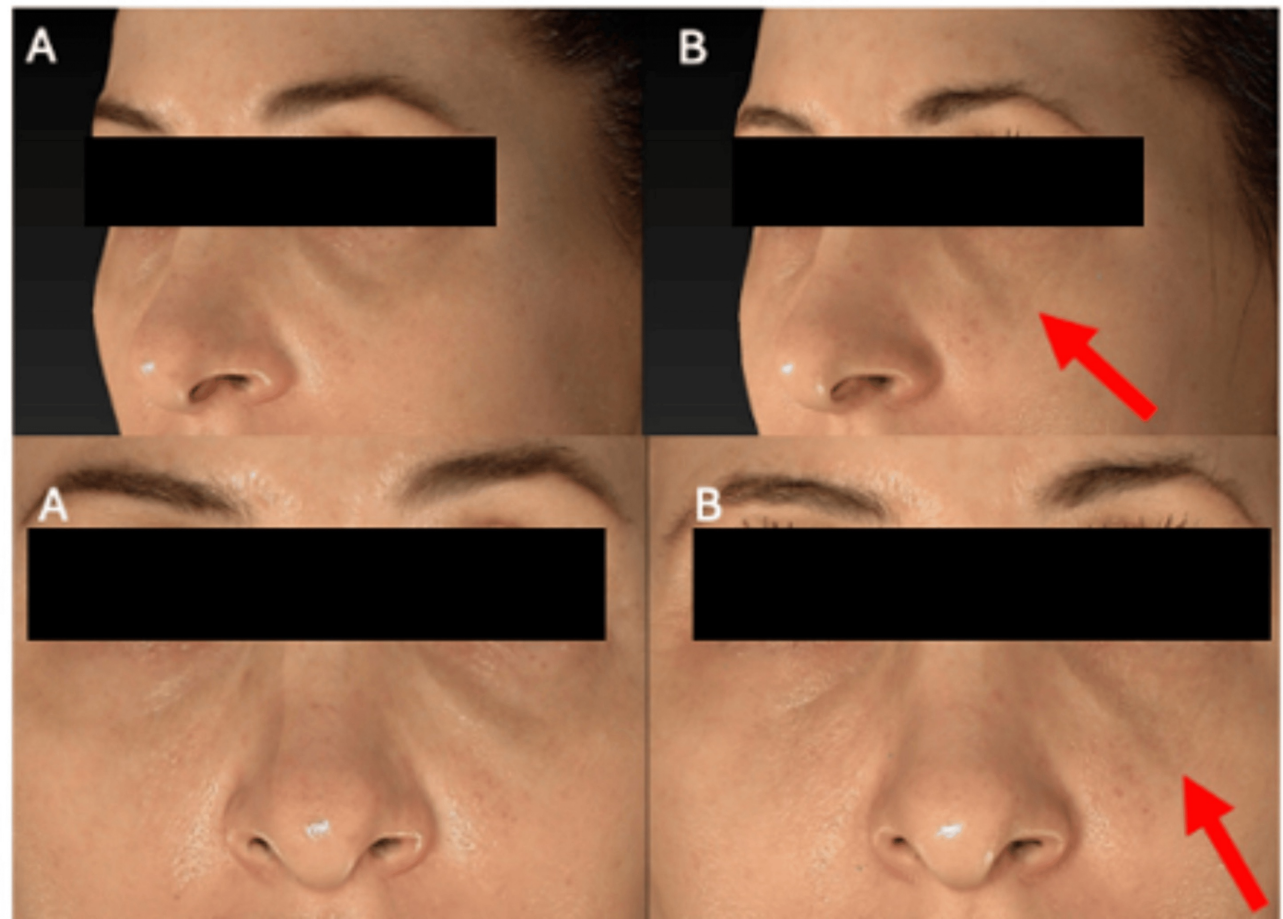
Post-hyaluronidase syndrome. A 31-year-old female patient was treated with 500 IU of hyaluronidase (PB3000, Pbserum, Madrid, Spain) to remove the previous hyaluronic acid on the left infraorbital side. (A) Before treatment photos, (B) after five days of the treatment. The red arrow indicates where the skin has lost volume, changed coloration, and experienced skin atrophy.

While hyaluronidase has a high effectiveness profile in resolving HA-related complications, concerns have recently arisen regarding undesirable outcomes after its application, including pain, irreversible volume loss, and adverse changes in skin appearance. This condition is known as "post-hyaluronidase syndrome" (Figure 1), which can manifest as Hollowing and volume loss, reduced skin elasticity, and skin pigmentation that worsens the appearance compared to pre-treatment [13]. Written informed consent to include all the images in the published article was obtained from the patients.

This condition is reported in approximately 18% of hyaluronidase applications [13]. However, this syndrome's exact mechanism of action is not yet fully understood, and some authors suggest it may be related to the volume and duration of the previously injected filler [13,14]. Enzymatic saturation may occur, replacing HA in the extracellular matrix. Repeated hyaluronidase applications can cause enzymatic saturation, negatively impacting skin quality in the affected area [10]. This risk is particularly significant in the periocular region, where the skin is thinner and more sensitive to volume loss and changes in the extracellular matrix. External factors such as pH, ambient temperature, and tissue type can also influence this risk [15].

**Figure 2:**
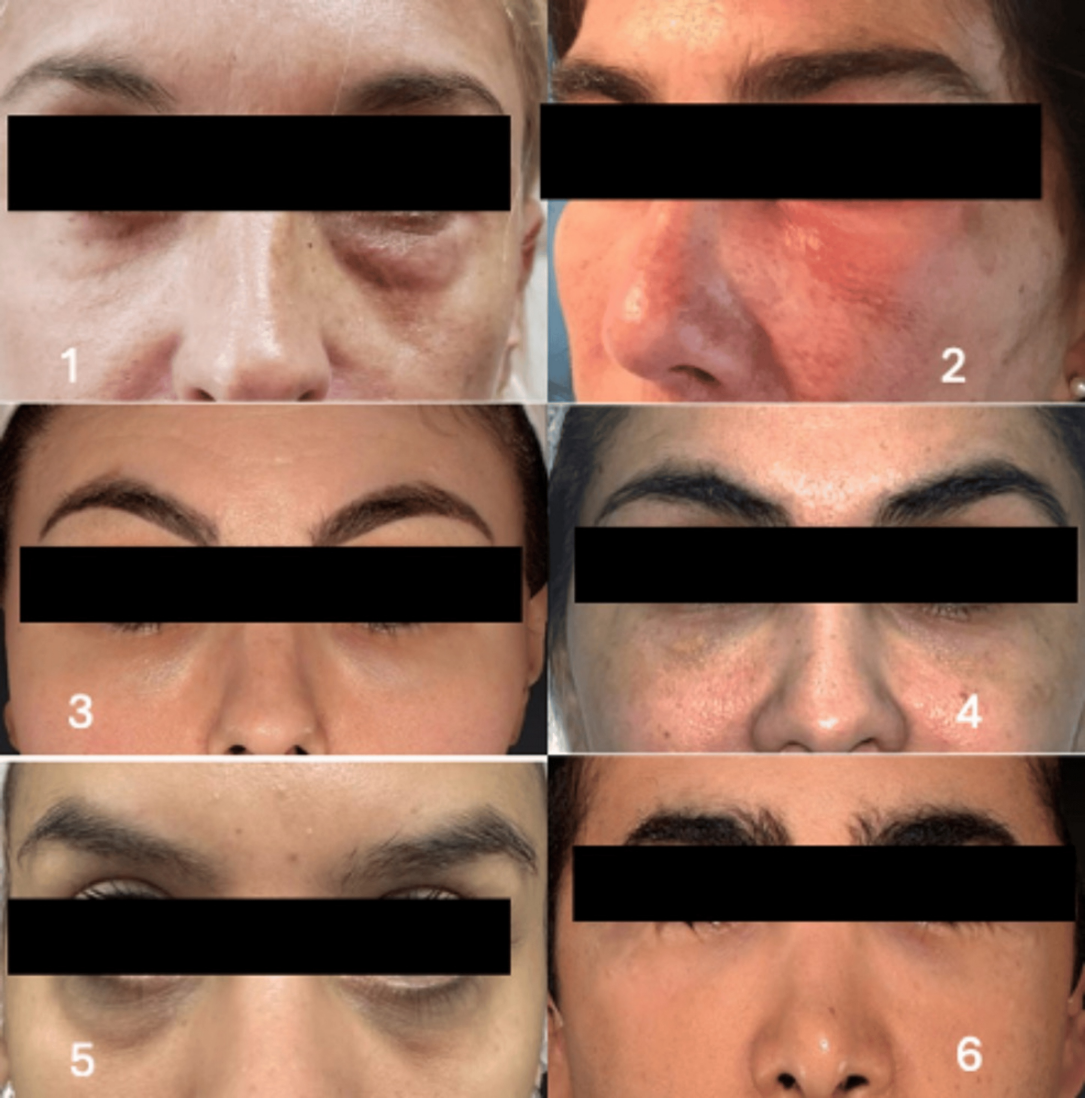
Before treatment photos from all patients. Edema is shown in all six patients (1-6) due to the application of HA at the tear trough level to enhance the periocular area. Patient #1: A 47-year-old female patient treated with Juvéderm Volbella (Vycross-Allergan, Irvine, CA); 0.3 ml was applied on each side, and edema remained two months later. Patient #2: A 50-year-old female patient treated with Juvéderm Volite (Vycross-Allergan, Irvine, CA); approximately 0.25 ml was applied on each side, presenting edema three months later. Patient #3: A 37-year-old female patient treated with Juvéderm Volbella (Vycross-Allergan, Irvine, CA); 0.2 ml was applied on each side, with edema present one month later. Patient #4: A 42-year-old female patient whose specific HA treatment was unknown; edema was observed four and a half months later. Patient #5: A 30-year-old female patient whose specific HA treatment was unknown; edema was present two months later. Patient #6: A 42-year-old male patient treated with Juvéderm Volite (Vycross-Allergan, Irvine, CA); approximately 0.35 ml was applied on each side, and edema was noted five months later.

We present a case series of six patients who exhibited periocular edema following injections of HA fillers, specifically Juvéderm Volbella (Vycross-Allergan, Irvine, CA) in patients 1 and 3 and Juvéderm Volite (Vycross-Allergan, Irvine, CA) in patients 2 and 6 (see Figure 2). Patients 4 and 5 were unable to identify the specific HA option administered. Written informed consent to include these images in the published article was obtained from the patient(s).

Desiree Castelanich conceptualized the technique in her private clinic in Buenos Aires, Argentina, where aesthetic medicine procedures are performed. She collaborated with Andrea Marcela Parra, an oculoplastic surgeon, and Luis Alberto Parra, an aesthetic physician. Together, the three practitioners treated the first three patients in Buenos Aires.

The remaining three patients were treated in various cities in Colombia by specialists who replicated the technique, adhering to the same dilution and application protocol.

All patients were classified as non-serious adverse events due to late-onset edema, and we applied Hirmand’s classification system [16], Teoxane Infraorbital Hollows Scale (TIOHS) [17], and O’Mahoney’s photo numeric scale [18] to evaluate the cosmetic outcome two months after management.

Hyaluronidase was recommended for the skin around the lower eyelid. However, using hyaluronidase in this area can lead to significant skin quality deterioration, including atrophy and changes in skin color, all of which are symptoms associated with "post-hyaluronidase syndrome” [13]. To mitigate this issue, we have developed an alternative treatment protocol that combines high-dose hyaluronidase with collagenase.

Description of technique

A comprehensive knowledge of the peri-orbicular anatomy is essential to deliver a safe treatment. The anatomy involves the skin measuring less than 1 mm with minimal subdermal fat [19,20]. The excessive swelling after injection in this area is believed to be due to tight ligament connections and restricted lymphatic drainage [20].

After a very detailed review of the location of edema in each patient, we decided to perform a combination of hyaluronidase with collagenase. ​​​​​​Diluted hyaluronidase and collagenase* *can be mixed as follows: (i) Start by diluting 1,500 IU of hyaluronidase (PB3000, PBSerum, Madrid, Spain) in 1.5 mL of 0.9% sodium chloride; (ii) dilute collagenase GH PB220 (Pbserum, Madrid, Spain) in 1.5 mL of standard saline solution containing 0.9% sodium chloride; (iii) mix 1.5 mL of hyaluronidase with 0.5 mL of collagenase to make 2 mL after dilution; (iv) apply this 2 mL combination to the area where the skin's appearance is targeted for improvement after the HA application. Depending on each patient's dermal thickness, you can administer it using a 27G x 50 mm or 25G x 50 mm cannula.

Chlorhexidine was used to cleanse the skin, and it was then left to air dry. The procedure was performed in small amounts using retrograde movements to cover the entire area, and one session was performed for all patients.

## Results

**Figure 3:**
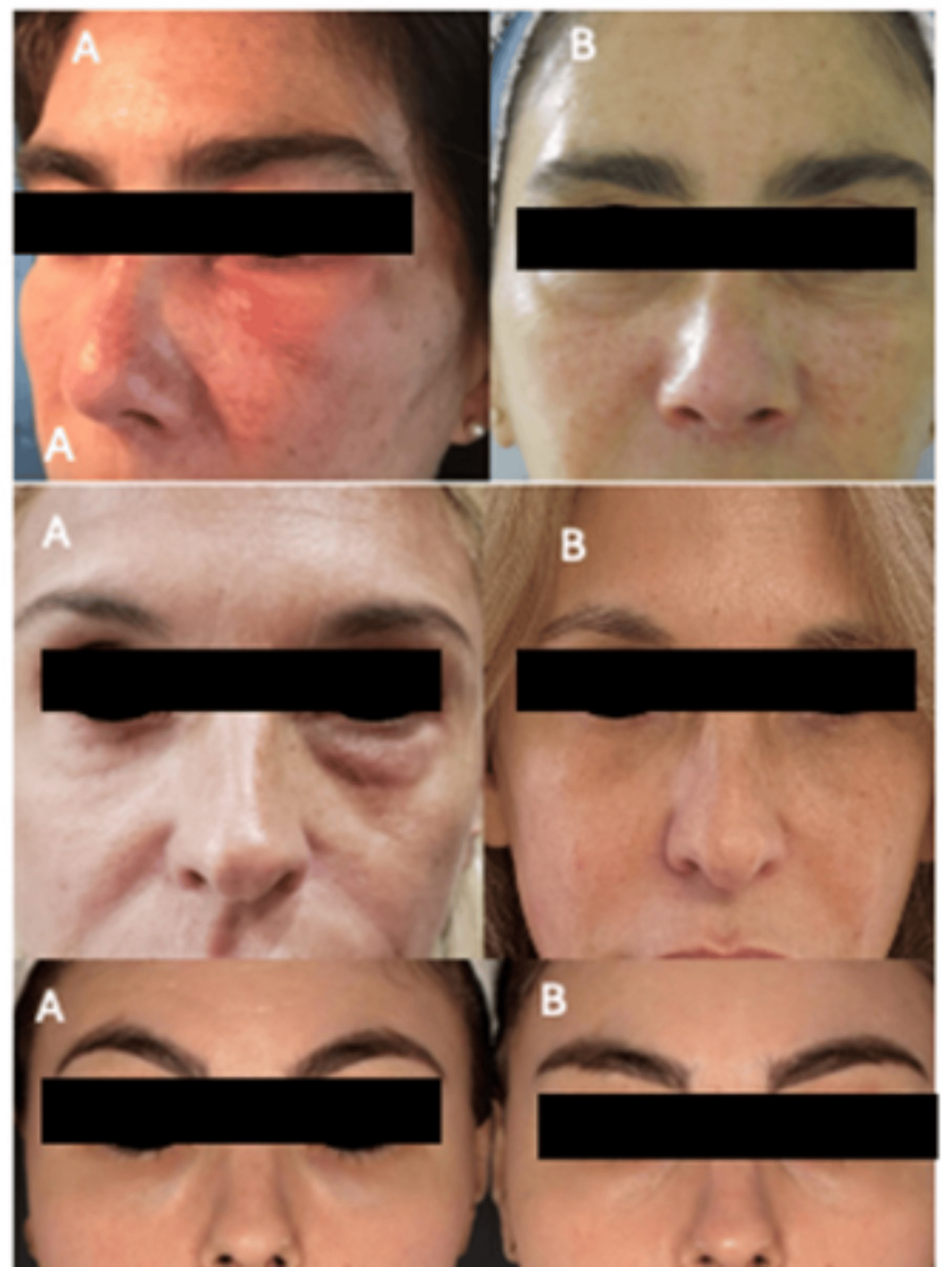
Follow-up photos for patients 1, 2, and 3. The photos show the before-and-after protocols for patients 1, 2, and 3 treated with the hyaluronidase + collagenase protocol; (A) before, and (B) two months after the treatment.

**Figure 4:**
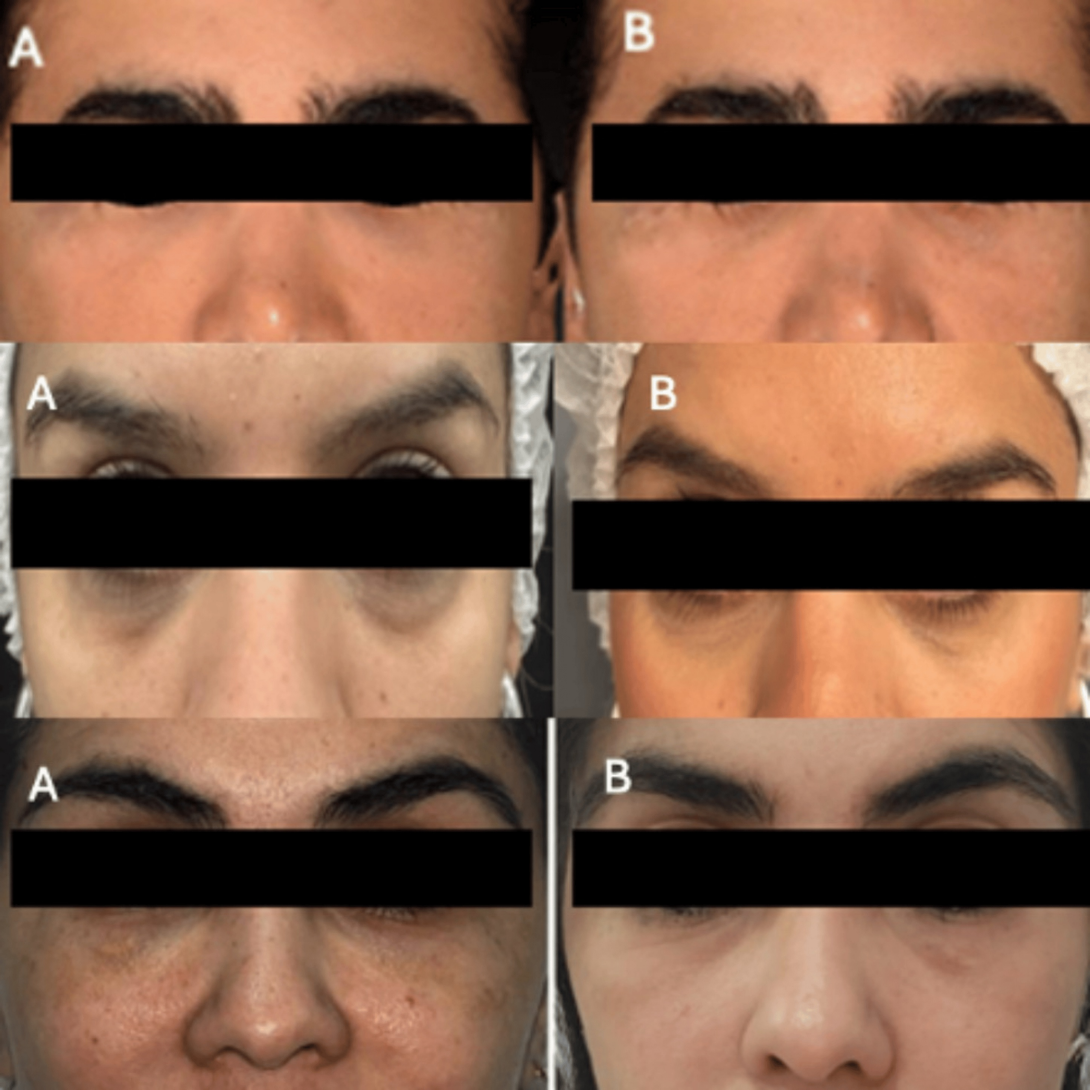
Follow-up photos for patients 4, 5, and 6. The photos show the before-and-after protocols for patients 4, 5, and 6 treated with the hyaluronidase + collagenase protocol; (A) before, and (B) two months after the treatment.

Two months after the enzymatic treatment (Figures 3, 4), no additional application of hyaluronidase was necessary. The results were satisfactory and aesthetically pleasing for the area, with no visible edema, loss of volume, or changes associated with post-hyaluronidase syndrome. Written informed consent to include these images in the published article was obtained from the patient(s).

The outcomes were evaluated using Hirmand’s classification system [16], the Teoxane Infraorbital Hollows Scale (TIOHS) [17], and O’Mahoney’s photo-numeric scale [18] to evaluate changes in the patients, and these results are presented in Table 1.

**Table 1:** Before and after Hirmand’s classification system [16], Teoxane Infraorbital Hollows Scale (TIOHS) [17], and O’Mahoney’s photo numeric scale [18] to evaluate the cosmetic outcome two months after management.

Patient	Before Hirmand’s classification system [16]	After Hirmand’s classification system [16]	Before Teoxane Infraorbital Hollows Scale (TIOHS) [17]	After Teoxane Infraorbital Hollows Scale (TIOHS) [17]	Before O’Mahoney’s photo numeric scale [18]	After O’Mahoney’s photo numeric scale [18]
1	Class III	Class II	4	2	8	3
2	Class III	Class I	5	1	8	4
3	Class II	Class II	3	2	5	3
4	Class II	Class I	3	2	5	2
5	Class III	Class I	4	2	7	3
6	Class II	Class I	3	1	5	2

Statistical analysis was conducted using IBM SPSS Statistics, version 30.0 (IBM Corp., Armonk, NY, USA). The Paired-Samples T-test was employed to evaluate and compare changes in Hirmand's classification system, Teoxane Infraorbital Hollows Scale (TIOHS) classification, and O'Mahoney's photo-numeric scale. The results indicate that all three interventions (Hirmand, TIOHS, and O'Mahoney) resulted in a statistically significant improvement in scores when comparing post-intervention to pre-intervention measurements (P < 0.05 for all scales and classifications). The effect size varied among the interventions, with O'Mahoney demonstrating the most substantial effect. The Hedges correction provided a more precise effect size estimate by accounting for sample size, resulting in 0.89 for Hirmand's, 1.3 for TIOHS, and 1.2 for O'Mahoney (Table 2). Consequently, the pre-and post-intervention results demonstrated a statistically significant difference in the aesthetic appearance of the patients' faces following treatment, as determined by four blinded evaluators who assessed the photographs.

**Table 2:** Effect sizes of paired samples. Results show all interventions (Hirmand, TIOHS, and O'Mahoney) significantly improve scores from ‘Before’ to ‘After’ (P < 0.05). Effect sizes differ, with O'Mahoney having the largest. Hedges correction, accounting for sample size, estimates effect sizes as 0.89 for Hirmand, 1.3 for TIOHS, and 1.2 for O'Mahoney. a: The denominator used in the estimation of effect sizes is the sample standard deviation of the mean difference, along with a correction factor provided by the Hedges correction. Hirmand’s classification system [16], Teoxane Infraorbital Hollows Scale (TIOHS) [17], and O’Mahoney’s photo numeric scale [18].

		95% confidence interval of difference		P value	
		Inf.	Sup.		Hedges correction^a^
Par 1	Before_Hirmand - After_Hirmand	.377	1.957	.013	.895
Par 2	Before_TIOHS - After_TIOHS	.850	3.150	.007	1.303
Par 3	Before_O’Mahoney’s - After_O’Mahoney’s	2.399	4.601	< .001	1.247

## Discussion

Injectable fillers are gaining popularity but may require dissolution for various reasons, primarily due to swelling or the Tyndall effect in the periorbital area. Distinguishing between overfill and swelling can be challenging. However, it is generally accepted that swelling is more pronounced in the morning, indicating edema caused by the filler's hydrophilic properties and daily fluctuations in body fluid distribution [13].

Wilde et al. (2024) first described "post-hyaluronidase syndrome," which involves hollowing and loss of volume, decreased skin elasticity, or skin pigmentation exceeding the appearance before the initial treatment [13]. According to their data, this syndrome could be found in 18% of patients who received hyaluronidase [13]. These findings were already evident to the authors in previous applications of hyaluronidase to remove HA previously positioned in the periocular area. Despite using low doses (approximately 500 IU of hyaluronidase per treatment), patients presented irreversible loss of natural volume and skin quality in the area (Figure 1).

After this experience, we evaluated a method to prevent post-hyaluronidase complications when removing HA from the periocular area. According to scientific literature, hyaluronidase can manage various complications and restore the effects of injected fillers. Recent studies have described the successful treatment of epidermoid cysts with recombinant hydrolytic enzymes by mixing hyaluronidase with other components [21].

Based on this evidence, we combined hyaluronidase with collagenases GH PB220 (Pbserum, Madrid, Spain). These collagenases break down collagen and offer benefits beyond their primary function, including promoting dermal cell regeneration, protecting against cell death and inflammation, and accelerating wound healing [22]. Bacterial-origin collagenases show significant effectiveness in breaking down collagen, promoting granulation tissue development, reducing inflammation, aiding epithelial cell regrowth, and speeding up wound healing. This highlights collagenase's potential in skin rejuvenation, scar management, and surgery preparation [21,23].

Varani et al. and similar literature suggest that dermal fibroblasts are connected to undamaged collagen fibrils and apply pulling forces to maintain standard cell structure and mechanical tension [24]. With aging, collagen fibrils become fragmented, leading to decreased fibroblast binding and reduced mechanical stability. These changes hinder fibroblasts' spreading, mechanical forces, and functions [24,25]. A critical feature of dermal fibroblasts in aged skin is diminished spreading and interaction with collagen fibrils, leading to a collapsed appearance. The cells deviate from their usual elongated spindle-like shape and become shorter, taking on a rounded and collapsed form [24].

Our technique represents a shift in the understanding of hyaluronidase usage. We propose administering a significantly higher dose in a single session, combining 1,500 IU of hyaluronidase (hyaluronidase PB3000, Pbserum, Madrid, Spain) diluted in 1.5 ml of 0.9% normal saline with 0.5 ml of a prepared dilution of collagenase GH PB220 (Pbserum, Madrid, Spain). We invite injectors to consider this combination to stimulate fibroblastic activity, thereby preventing skin retraction by altering fibroblast tension and the structure of the extracellular matrix, which can trigger the recovery of the entire extracellular matrix.

Four blinded assessors with extensive injectable experience reviewed before-and-after photographs using three scales to measure aesthetic improvement. Although these scales do not specifically assess complications, we suggested they evaluate the overall enhancement of these cases. The changes were then compared statistically using the paired t-test and Hedges' correction to determine effect size and correct for estimation bias. These tests indicated a statistically significant decrease in adverse outcomes, with a positive mean difference in both cases, suggesting improvement after treatment. According to the confidence interval results and Hedges' correction in a small sample, we can conclude that the management successfully enhanced the aesthetic appearance of the treated patients.

A fundamental limitation of our study is the small number of patients treated; the technique has only been tested on six patients. Despite this limitation, the results appear promising and suggest that practitioners in various countries could easily replicate this approach regardless of patients' skin types. This is one of the main strengths of our study, as three patients were treated in Argentina and three in Colombia, demonstrating the technique's adaptability and replication potential. In the future, we hope to continue accumulating data to validate these findings further.

## Conclusions

We propose a novel technique combining 1,500 IU of hyaluronidase (hyaluronidase PB3000, Pbserum, Madrid, Spain) with collagenase GH PB220 (Pbserum, Madrid, Spain) in the same syringe to correct late-onset edema in the periocular region after HA applications. This approach demonstrates a high safety profile in the four patients studied. It provides evidence of improved edema in a single session using an average of 1,500 IU of hyaluronidase without triggering symptoms of post-hyaluronidase syndrome.

Statistical analysis revealed a significant improvement in aesthetic scores, with a positive mean difference observed in the paired t-test results (P < 0.05) across all scales used. The Hedges correction indicated effect sizes of 0.89 for Hirmand, 1.3 for TIOHS, and 1.2 for O'Mahoney, further supporting the efficacy of our approach. Although the study's limited sample size may preclude definitive conclusions, exploring alternative solutions for managing HA complications that require fewer applications while preserving the extracellular matrix and skin quality is essential. This study may pave the way for future histological and cellular function analyses to confirm the product's positive effects.
